# Correlation between calcium, water contents and ultrasonographic appearance of atherosclerotic lesions of carotid artery lesions

**DOI:** 10.1515/tnsci-2020-0115

**Published:** 2020-08-24

**Authors:** Béla Fülesdi, Szabolcs Farkas, Zoltán Gyöngyösi, Péter Siró, Dániel Bereczki, József Bacsó, László Csiba

**Affiliations:** Department of Anesthesiology and Intensive Care, Faculty of Medicine, Division of Neurointensive Care and Neuroanesthesia, University of Debrecen, H_4032 Debrecen, Nagyerdei krt. 98, Hungary; Department of Neurology, University of Debrecen, Debrecen, Hungary; Department of Neurology, Semmelweis University, Budapest, Budapest, Hungary; Insitute of Nuclear Research, Hungarian Academy of Sciences, Debrecen, Hungary

**Keywords:** Ca and water contents, carotid plaque, B-mode ultrasound, ultrasonic tissue characterisation

## Abstract

**Background:**

We tested the hypothesis whether there is a correlation between the echogenicity and calcium and water contents of carotid plaques.

**Patients and methods:**

Ninety carotid befurcations from 45 deceased patients were removed during autopsy. Thirty-four plaques were categorized as homogenous echolucent (HEL), homogenous echogenic (HEG) and heterogenous (HE) plaques based on premortem B-mode image. Water content was expressed in % of wet weight. Ca was determined by proton-induced X-ray emission and expressed in ppm. Relative optical density of the B-mode images was analyzed offline using a computer program.

**Results:**

HEL plaques had lower Ca content (medians and IQRs: 6,145 [4,465–6,536 ppm]) compared to HEG (74,100 [15,300–1,44,500−ppm]), *P* ≤ 0.001). HE plaques showed an intermediate calcium content (7,310 [4,840–9,920 ppm]) that was statistically not different from echolucent plaques. Water content of HEG plaques was statistically not different from HEL and HE (HEG:53.5 [35.5–64%], HEL: 73.5 [69.7–78.5%], HE: 70.6 [67.4–73.9%]). HEG plaques had the highest relative optical densities (196 [188–217%]). HEL and HE had similar relative optical densities (HEL: 176 [164–187%], HE: 164 [144–188%], respectively). A significant positive correlation was found between the Ca content and relative optical density of plaques.

**Conclusions:**

Echogenicity of carotid plaques increases along with their calcium content. Water content may be an important factor in differentiation of different plaques.

## 1 Introduction

Atherosclerosis is a diffuse pathological process characterized by deposition of lipid and other blood-borne material within the arterial wall of almost all vascular territories [1]. The main components of the atherosclerotic plaques are (a) connective tissue extracellular matrix, including collagen, proteoglycans and fibronectin elastic fibers; (b) crystalline cholesterol, cholesteryl esters and phospholipids; (c) cells such as macrophages, T lymphocytes and smooth muscle cells [2]. It has been shown that atherosclerotic lesions contain calcium phosphate deposits, as well [3]. Vascular calcification is evidently a generalized active process arising in the areas of chronic inflammation, is intimately involved with the inflammatory condition of the intima and now believed to be a key process in atherogenesis [4]. Atherosclerosis (and its progression) is the key substrate for the development of arterial vascular symptoms [5]. Various authors outlined the complexity of the relationship between inflammation, angiogenesis and intraplaque hemorrhages in plaque progression and destabilization [6].

For a long time, it was believed that the degree of carotid stenosis is of paramount importance, causing hemodynamic strokes [7], but later studies proved that even low-grade carotid stenosis may raise the risk of ischemic cerebrovascular events [8,9]. B-mode ultrasonography generates high-resolution images of carotid plaques providing information about their location, extent, contour, composition as well as the amount of associated stenosis. As the absorption of ultrasound depends on the different acoustic impedance of tissue components (e.g., collagen, tissue water, lipids, blood, calcification), the echoreflexion of an atherosclerotic lesion is determined by the homogeneity or heterogeneity of the tissue structure [10]. Based on the ultrasound images, Gray-Weale et al. [11] have classified the atherosclerotic plaques in four types: type 1 is predominantly echolucent, type 2 is mainly echolucent with small areas of echogenicity, type 3 is predominantly echogenic with small areas of echolucency, and type 4 is uniformly echogenic. The recent decade has seen a huge development in better understanding the association between plaque echostructure and stroke symptomatology [12]. In a recent study, it was demonstrated that 43.2% of symptomatic plaques are echolucent in comparison to 24.6% of asymptomatic plaques (*p* = 0.02), whereas 40.9% of symptomatic plaques are echogenic in comparison to 67.2% of asymptomatic plaques [13].

Some authors have tried to compare the ultrasonic tissue characterization with biochemical factors that might play a role in echoreflexion [14]. However, only few studies have been performed to find the relationship between the Ca and water content and the echogenicity of occlusive atherosclerotic lesions [15,16].

In this study, we have used computer-assisted B-mode ultrasonography image analysis to measure the echostructure of carotid atherosclerotic atherosclerotic lesions and attempted to compare their calcium and water contents. The aim of the present study was to check whether a correlation exists between the echogenicity and calcium as well as the water content of carotid plaques with different echostructures. According to our hypothesis, testing the hypothesis that both the calcium and water contents of carotid plaques are the main factors in determining that echogenicity could increase the amount of information about plaque composition and stability gained by the B-mode ultrasonography.

## 2 Materials and methods

### 2.1 Patients

The subjects of this investigation were 45 critically ill patients admitted to the Stroke Unit of the Department of Neurology, University of Debrecen, with the diagnosis of acute ischemic stroke, between the time period of November 2012 and February 2014. Subjects underwent B-mode carotid ultrasound examination as a part of the routine diagnostics. All patients had acute ischemic cerebrovascular lesions confirmed by computer tomography. Confirmed carotid stenosis on the B-mode ultrasound imaging were the main inclusion criteria for our study.

**Ethical approval:** The research related to human use has been complied with all the relevant national regulations, institutional policies and in accordance with the tenets of the Helsinki Declaration, and has been approved by the Ethics Committee at University of Debrecen (RKEB-TUKEB 218/2012 OEC).

**Informed consent:** Informed consent has been obtained from all individuals included in this study.

Phases of the study

1. B-mode ultrasonography in 45 moribound patients admitted with the diagnosis of acute ischemic stroke as a part of the routine diagnosis. Storage and categorization of the lesions (predominantly echolucent, mixed echolucent, predominantly echogenic plaques) seen on the B-mode image (see detailed description below).
2. Removal of the carotid bifurcations and sampling the lesions that were seen on the B-mode image.
3. Biochemical analysis of tissue samples for determining the Ca and water contents (see detailed description below).
4. Off-line analysis of the relative optical density of carotid plaques (see detailed description below).

### 2.2 Ultrasound examinations

Ultrasound examinations were performed with a Hewlett-Packard Sonos Ultrasound equipped with a 10 MHz linear-array transducer. Common and internal carotid arteries and bifurcations were scanned longitudinally by the B-mode method for the presence of atherosclerotic plaque. In order to avoid variability in image acquisition between subjects, ultrasound gain was stepwise adjusted in each individual measurement from a bright image to the darker direction until the point where the appearance of the flowing blood just turned into total black.

According to the modified proposal of Gray-Weale [11], visual analysis and classification of plaque echolucency on the B-mode images were performed using the following classification:

Type 1: predominantly echolucent

Type 2: mixed echolucent/echogenic

Type 3: predominantly echogenic

All scans were performed by two experienced neurosonologists (BF and PS), who were not involved in the later phases of analysis (densitometric analysis, postmortem tissue sampling and analysis).

### 2.3 Tissue sampling

Sampling of the tissues occurred during the routine autopsy following the patients’ death within 24 h post mortem. Carotid bifurcations were removed, dissected longitudinally and examined macroscopically. Removal and preparation of the samples for further analysis were performed by two independent members of the team (SF and ZG). Calcium and water contents have been measured only on those bifurcations where macroscopic lesions were unequivocally identical to the lesions observed on the ultrasound images. For matching images to histology sections, we have used the relative distance of plaque from the common carotid bifurcation as a landmark, expressed in millimeters. This was carried out by a software option of the duplex scanner, resulting in identifying of the lesion location on the basis of the *in vivo* measurement.

### 2.4 Analysis of tissue samples

During sampling, an attempt was made to remove plaques unimpaired.

#### 2.4.1 Determination of water content

After excision, tissue samples were weighed on a chemical balance to obtain wet weight (*W*) with 0.0001 g precision. The samples were then dried at 100°C for 24 h and reweighed to obtain dry weight (*D*). Water content was calculated as follows: 100(*W* − *D*)/*W* and were expressed in percents.

*Proton-Induced X-Ray Emission* (PIXE) measurements were carried out at the Debrecen microprobe facility installed on the 0° beam line of the 5 MeV Van de Graaf accelerator at the Institute of Nuclear Research of the Hungarian Academy of Science. After pretreatment, dried samples were bombarded by a proton beam of 2 MeV. The characteristic X-ray spectra of the samples were measured by a silicium-semiconductor X-ray spectrometer both qualitatively and quantitatively. The PIXE analysis gives information about the absolute amounts of the elements present within the beam spot which, however, cannot be directly converted to tissue concentrations. Hence, the PIXE spectra were analyzed by personal computer using XRF-BIO, a self-made program, estimating Ca concentrations corresponding to parts per million (ppm). For the calibration procedure, NBS and IMEA standards were used. Tissue analysis was performed by the same physicist (JB) who had previous experience in the analysis of vessel segments [15].

### 2.5 Densitometric measurements

All B-mode images were stored and were later off-line processed with the software program Image-Pro Plus version 1.2 for Windows (Media Cybernetics, Rockville, USA) as suggested by the methodology by Grønholdt et al. [17]. Any area on the screen could be outlined via computer mouse, and an average optical density of the respective area could be measured by the image analysis system. Thus, an averaged relative optical density of the region of interest (normal vessel wall and plaque) was determined. The optical density of the circulating blood, considered 100%, was used as a reference value for densitometric measurements. Therefore, the optical density of plaques was compared to that of the blood, thus making possible the objective quantification of echogenicity and its comparison with other examined parameters (calcium and water contents of atherosclerotic lesions).

### 2.6 Statistical analysis

The normality of variables was checked by the Shapiro–Wilk test. Calcium, water and optical density were not normally distributed; therefore, median and quartile ranges were calculated. For any comparison where these data were involved, Wilcoxon Sum-of-Ranks (Mann–Whitney) test was used for comparing two unmatched samples. The Spearman’s rank-order correlation was used to describe the relationship between variables with nonnormal distribution. Statistical significance was assumed if *P* < 0.05. Statistica for Windows version 5.5 (StatSoft, Tulsa) was used for data analysis.

## 3 Results

Ninety bifurcations of 45 deceased patients were removed during autopsy. There were 29 male patients (64.4%) and 16 females (35.6%), with a mean age of 65.1 (±5.6) years. Clinical characteristics are summarized in Table 1. Based on the pathological diagnosis, the causes of death of the patients were pneumonia (aspiration or bronchopneumonia *n* = 28), myocardial infarction (*n* = 7), pulmonary edema (*n* = 4) or pulmonary embolism (*n* = 6).

**Table 1:** Main clinical characteristics of the patients

Characteristic	Value
Age (years)	65.1 (±5.6)
Gender (F/M)	16/29
Hypertension, *n* (%)	33 (73)
Diabetes mellitus, *n* (%)	14 (31)
Smoking (Y/N)	28/17
Atrial fibrillation, *n* (%)	6 (13)
NIH score at admission, *n* (%)	
0	2 (4)
1–4	3 (7)
5–8	15 (33)
9–12	17 (38)
13–17	8 (18)

Thirty-four plaques (16 homogeneous and 18 heterogeneous) were diagnosed, categorized by the B-mode ultrasound during the clinical treatment phase. After the death of the patients, the identical carotid bifurcations were removed and dissected during autopsy. All plaques were located at the proximal part of the internal carotid arteries. As described earlier, based on their ultrasound appearance, lesions were grouped into homogenous plaques with predominantly echolucent, or with predominantly echogenic structure and heterogenous plaques with mixed echogenicity/echolucency.

### 3.1 Ca content

**Figure 1:**
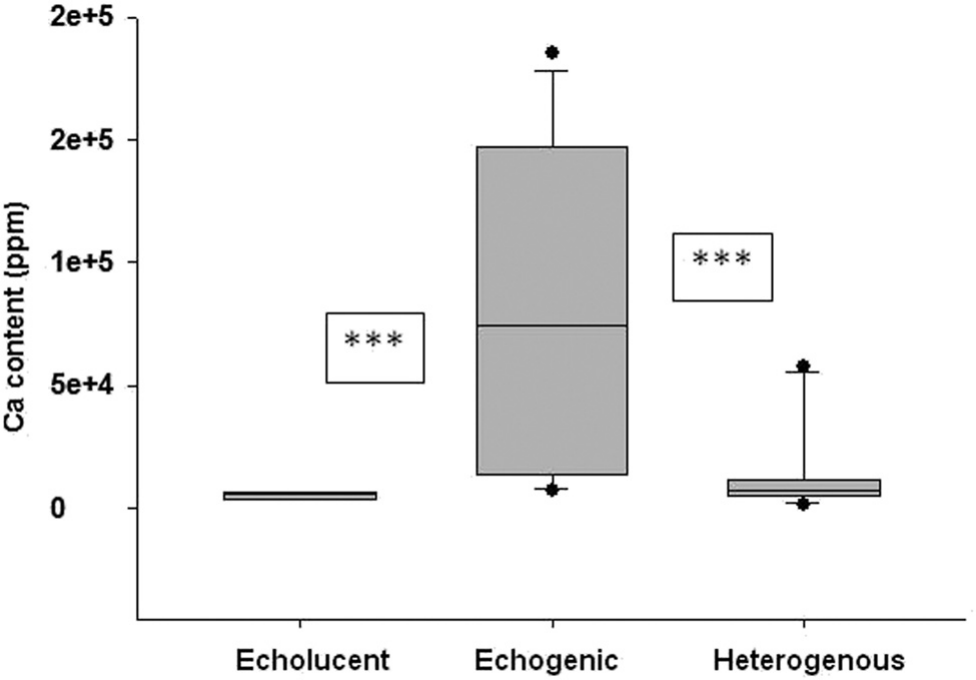
Ca content of echolucent, echogenic and heterogenous plaques. Data are presented as medians, IQRs, minimum and maximum values. *** represents *p* < 0.001 NS represents nonsignificant differences.

Echolucent plaques had significantly lower Ca content (medians and IQRs: 6,145 [4,465–6,536 ppm]) compared to echogenic plaques (medians and IQRs: 74,100 [15,300–1,44,500 ppm]), respectively, *P* ≤ 0.001). Heterogeneous plaques showed an intermediate calcium content (medians and IQRs: 7,310 [4,840–9,920 ppm]) that was statistically not different from echolucent plaques. Data are depicted on Figure 1.

**Figure 2:**
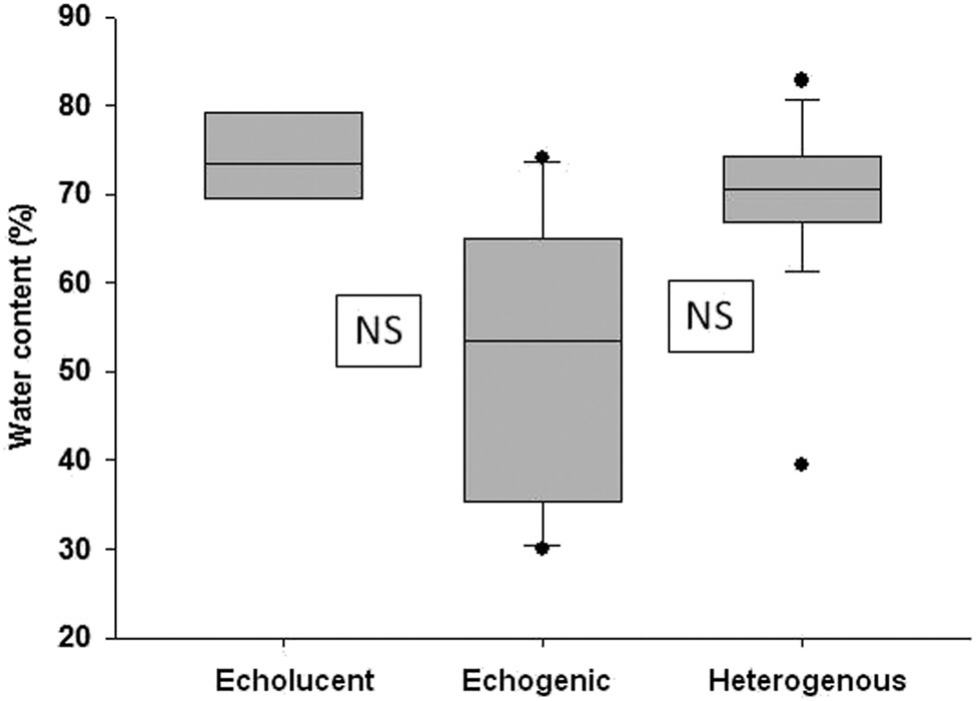
Water content of echolucent, echogenic and heterogenous plaques. Data are presented as medians, IQRs, minimum and maximum values. NS represents nonsignificant differences.

Water content in echogenic plaques was statistically not significantly different from that of echolucent and heterogenous plaques (medians and IQRs echogenic: 53.5 [35.5–64%], echolucent: 73.5 [69.7–78.5%], heterogenous: 70.6 [67.4–73.9%], respectively). It has to be mentioned that there was a tendency of echogenic plaques to have lower water content, but the differences did not reach the level of significance, probably due to the sample size. Data are summarized in Figure 2.

A significant inverse correlation has been found between water and Ca contents of plaques (Spearman’s correlation coefficient: −0.56, *P* ≤ 0.0001), i.e., the higher was the Ca content, the lower was the water content of plaques.

### 3.2 Optical density measurements

**Figure 3:**
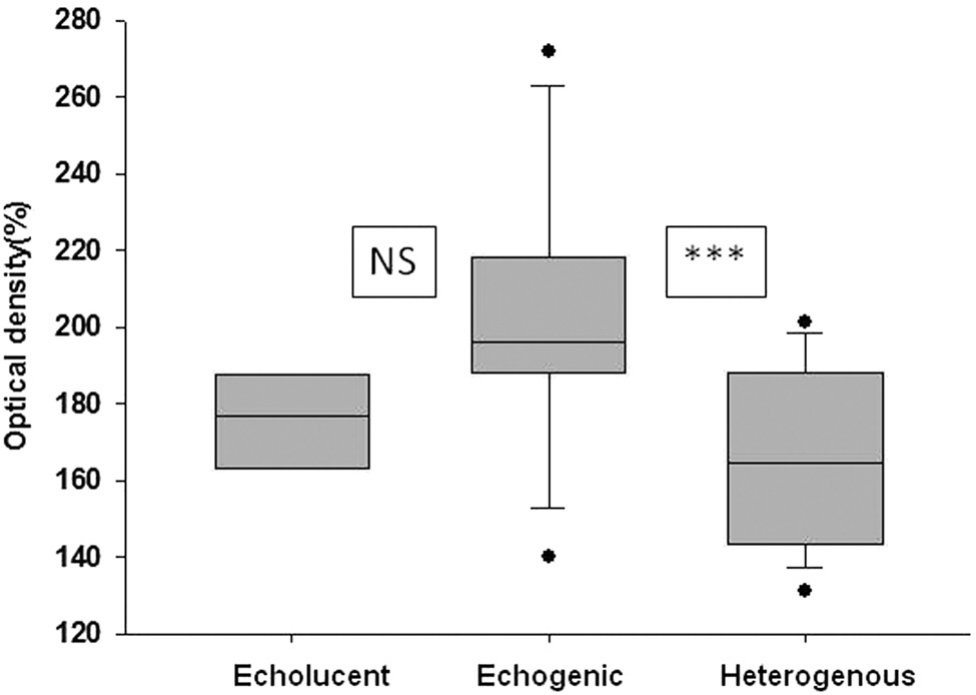
Relative optical density of echolucent, echogenic and heterogenous plaques. Data are presented as medians, IQRs, minimum and maximum values. *** represents *p* < 0.001, NS represents nonsignificant differences.

Echogenic plaques had the highest relative optical densities (medians and IQRs echogenic: 196 [188–217%]). Echolucent and heterogeneous plaques had similar relative optical densities (echolucent: 176 [164–187%] and heterogenous: 164 [144–188%], respectively) (Figure 3).

### 3.3 Correlation between calcium, water contents and relative optical density in plaques

In general, the calcium content of different plaques significantly correlated with their relative optical density (Spearman’s correlation coefficient: 0.71, *P* = 0.001). The water content of different lesions and their optical densities were negatively correlated (Spearman’s coefficient = −0.36, *P* = 0.01).

## 4 Discussion

In the present study, we have found that echolucent carotid plaques on the B-mode ultrasound have lower Ca content but higher water content compared to echogenic plaques, whereas plaques with mixed (heterogenous) echogenicity are in the intermediate position with regard to Ca and water contents. When the optical density of different lesions was plotted against their Ca content, a significant positive correlation could be detected between the two parameters.

Previous histopathological studies revealed that echogenicity of the plaque is determined by its composition: hypoechoic heterogeneous plaques are associated with intraplaque hemorrhage (increased water content) and lipids, whereas hyperechoic homogeneous plaque is mostly fibrous or calcified. Elastin content has also been associated with echolucency [18,19]. The development of atherosclerosis is a complex process. The initial endothelial injury, initiated by the lipoprotein infiltration into the subendothelial space, is followed by inflammation, neoangiogenesis and repairing processes, which are involved in the pathogenesis of atherosclerotic plaques and their thrombotic complications [6,20]. The rupture of blood vessels in the wall of the plaque, secondary to neoangiogenesis, or blood entering from the lumen, secondary to fissuring of the plaque, is also an important factor in the atherosclerotic plaque growth, inflammation and destabilization [14,21]. Due to these processes, the water content of plaques changes during the progression of the atherosclerotic disease; however, its excessive presence could be an evidence of plaque instability. According to our results, the water content of plaques was lower than that of the normal vessel wall. Comparing homogeneous with heterogeneous plaques, we have found significantly higher water content at heterogeneous ones, which is related probably to the presence of intraplaque hemorrhages.

In our study, plaques were characterized by high calcium in comparison with normal vessel wall. Mineralization and lipid deposition are coincident in plaques [22,23]. The amount of calcification has been found larger in asymptomatic plaques by many authors; thus, it seems to have played an important role in plaque stabilization [24,25]. However, the role of calcium deposits to plaque vulnerability or stability is a matter of debate. On one hand, authors report that the location rather than the size of the calcification may play an important role. It has been demonstrated that calcification in the lipid pool does not increase fibrous cap stress when it is distant to the fibrous cap, while large areas of calcification close to the fibrous cap or calcification in the thin fibrous cap may lead to a high stress concentration within the fibrous cap, which may cause plaque rupture [26,27,28]. There are reports indicating that the proportion of calcified segments relative to the total plaque volume might be decisive. According to this concept, heavily calcified plaques (>45% of total plaque volume) demonstrate a strong predilection toward stability. As the calcified content increases up to a point, there are increased interfaces between the plaque contents leading to a possible increased risk of rupture. However, at a point where plaques become heavily calcified, the interface area between the stiffer and the more distensible plaque decreases, which may lead to a decreased risk of rupture [24]. What it comes down to is that both location and degree of the calcification may play an important role in plaque vulnerability, respectively, stability. In addition, plaque calcification and inflammation rarely overlap; furthermore, lipid-rich plaques are more inflamed than either calcified or collagen-rich plaques. These support the theory that calcification represents a late, burnt-out stage of atherosclerosis [29].

Computer-based densitometric analysis and mean pixel determination of carotid ultrasonic images offer the possibility of digitizing B-mode images, allowing enhanced differentiation of plaque composition; furthermore, they can be used to standardize images and make measurements of echogenicity objective [13,14]. However, some studies report that the value of computer-assisted ultrasonography, measuring the echogenicity of the entire plaque, in the determination of plaque composition is limited; even though, one of them noted that intraplaque hemorrhages are identifiable [30,31]. Furthermore, by evaluating plaque echogenicity using the computerized image analysis, authors have found adjacent but different cut-off points for the differentiation between symptomatic and asymptomatic plaques [32]. The reason for this is that the gray-scale median analysis figures represent the average for the whole arteriosclerotic area. Nevertheless, the value of the computer-assisted image analysis of the B-mode images can be increased by analyzing pixel distribution over certain regions of interest within the atherosclerotic lesion [33].

Our results show that homo- and heterogeneous plaques had higher calcium content and optical densities, explaining why plaques can be easier detected by the ultrasound. In spite of the fact that calcium accumulates continuously in the plaque, we have found no significant difference of calcium content and relative optical density between homogeneous and heterogeneous plaques, which could be explained by the complexity of intraplaque events during plaque ageing or by measuring the entire plaque area to express its relative optical density. It was not the objective of our study to define a threshold value of optical density for the distinction of different types of plaques. Our main purpose was to confirm the correlation between echogenicity and calcium and water contents in the atherosclerotic lesions. According to our results, calcium seems to be one of the major determinants of the optical density, with high calcium concentrations resulting in high optical densities, whereas water content has a definitive impact on plaque echogenicity.

What is the clinical implication of the results? In previous studies, different factors have been identified that may lead to an increased TIA and stroke risk in patients with hemodynamically nonsignificant carotid stenosis: the length of the stenosis along with a consequently increased cross-sectional area of the plaque, thin or ruptured fibrous cap, lipid-rich necrotic core, and plaque hemorrhage [34]. All these factors can be sensitively assessed by high-resolution ultrasonography. Biochemical analysis of the atherosclerotic lesions in parallel with ultrasonic may help in better understanding the natural history of plaque maturation process and improve the accuracy of ultrasonic tissue characterization.

We have to mention the limitations of our study. The most important limitation is related to the averaged relative optical density of the carotid lesion. In fact, pixel distribution would have provided better description of echogenicity of the lesions, pathological samples were taken from the macroscopic gross regions of the carotid arteries and an averaged water content and Ca content could be determined by further analysis as well. We believe that despite this limitation, these data may describe the nature of plaque maturation process.

Our conclusions are that [1] relative optical density (echogenicity) increases along with the calcium content, [2] and there is an inverse correlation between the water and calcium content of plaques, indicating that during the maturation process of the plaques, the water content decreases along with increasing calcification [3]. Calcium content may be the major factor in determining echogenicity of plaques. Ultrasonic tissue characterization may contribute to better understanding of the natural history of plaque maturation process and may help in differentiation between stable and unstable carotid plaques.
